# Characterization of donkey-meat flavor profiles by GC–IMS and multivariate analysis

**DOI:** 10.3389/fnut.2023.1079799

**Published:** 2023-03-16

**Authors:** Limin Man, Wei Ren, Mengqi Sun, Yanrui Du, Han Chen, Huaxiu Qin, Wenqiong Chai, Mingxia Zhu, Guiqin Liu, Changfa Wang, Mengmeng Li

**Affiliations:** School of Materials Science and Engineering, School of Agricultural Science and Engineering, Liaocheng Research Institute of Donkey High-Efficiency Breeding and Ecological Feeding, Liaocheng University, Liaocheng, China

**Keywords:** donkey meat, flavor profile, GC–IMS, multivariate analysis, volatile compounds

## Abstract

The distinctive flavor compounds of donkey meat are unknown. Accordingly, in the present study, the volatile compounds (VOCs) in the meat from SanFen (SF) and WuTou (WT) donkeys were comprehensively analyzed by gas chromatography–ion mobility spectrometry (GC-IMS) combined with multivariate analysis. A total of 38 VOCs, of which 33.33% were ketones, 28.89% were alcohols, 20.00% were aldehydes, and 2.22% were heterocycles, were identified. Ketones and alcohols were significantly more abundant for SF than for WT, whereas aldehydes showed the opposite trend. The donkey meats from the two strains were well differentiated using topographic plots, VOC fingerprinting, and multivariate analysis. A total of 17 different VOCs were identified as potential markers for distinguishing the different strains, including hexanal-m, 3-octenal, oct-1-en-3-ol, and pentanal-d. These results indicate that GC–IMS combined with multivariate analysis is a convenient and powerful method for characterizing and discriminating donkey meat.

## 1. Introduction

Donkey husbandry is an important industry in China and has become instrumental in optimizing animal farming practices, enriching the number and types of animal products available to the consumer, meeting diversified consumption demands, increasing farmers’ incomes, and implementing rural revitalization (1). Donkey meat has gained increasing popularity because of its nutritional value and distinctive flavor. It is a tender, low-calorie meat with high protein and polyunsaturated fatty acid contents. Accordingly, it has become accepted as a high-quality meat, and it accounts for more than 80% of the economic benefits of donkey breeding and production (2–4).

Flavor is one of the most important factors of meat quality. Meat flavor is the result of the combination of volatile compounds (VOCs), which are sensed by smell and taste (5). Meat flavor directly affects sensory characteristics, food quality, and consumers’ purchase intention, and it is affected by the source, type, and processing of the meat (6, 7). More than 1,000 VOCs belonging to nine categories, including alcohols, aldehydes, esters, ketones, and acids, have been identified in meat and meat products (8). VOCs contribute to the characteristic flavor of meats and are therefore closely related to consumer preference (9). For instance, a large number of VOCs, including aldehydes, ketones, alcohols, carboxylic acids, and esters, have been detected in mutton, with 4-methyl octanoic acid and 4-ethyl octanoic acid playing major roles in mutton smell (10); while aldehydes are the main VOCs in donkey meat, as revealed by solid-phase microextraction–gas chromatography–mass spectrometry (SPME–GC–MS), with hexanal the most abundant volatile flavor compound (3, 11). Other SPME-GC–MS studies have shown that maltotriose, L-glutamate, and L-proline are the main contributors to the unique taste of donkey meat (12). GC–MS or gas chromatography–olfactometry–mass spectrometry (GC–O–MS) are commonly used to identify the VOCs in meat and meat products (13). However, these methods require complex sample pre-treatments (e.g., heating, distillation, and extraction) and long detection times, leading to untimely and inaccurate determination results (14).

Gas chromatography–ion mobility spectroscopy (GC–IMS), which has the advantages of rapid detection, stable results, and convenient operation without sample pre-treatment, is a new method to detect the VOCs of meat and meat products (15, 16). Using GC–IMS in combination with principal component analysis (PCA), beef, mutton, and chicken were distinguished with a classification accuracy of 98.3% (17). Furthermore, GC–IMS and statistical methods have been used to identify significant differences in the types and contents of VOCs in bacon from different pork breeds (18). In addition, the VOCs of Jinhua ham aged for different times and dry-cured pork with different salt contents have been analyzed based on GC–IMS and chemometrics analysis (19, 20). However, GC–IMS of VOCs combined with multivariate analysis has not been applied to establishing a methodology for the identification and analysis of donkey meat.

Accordingly, in the present study, the VOC profiles of donkey meat from SanFen (SF) and WuTou (WT) donkeys were comprehensively analyzed and compared by GC–IMS combined with multivariate analysis. Ultimately, our results facilitate a better understanding of the characteristic VOCs of donkey meat and provide a novel strategy for its authentication.

## 2. Materials and methods

### 2.1. Sample collection

A total of 12 healthy two-year-old Dezhou donkeys including 6 SF donkeys and 6 WT donkeys, with both containing 3 males and 3 females, which were obtained from a local farm in Liaocheng (Shandong, China). All donkeys were fed same diet and raised under the similar condition. Donkeys were transported to a local slaughter house (Shandong Dong’a Tianlong Food Co., Ltd., China) and killed according international standards (CAC/RCP 41-1993 and ISO/TS 34700: 2016) after starvation for 12 h. After slaughter, the longissimus dorsi (Between the 17th and 18th ribs) was acquired. The muscle was immediately frozen in liquid nitrogen and stored at −80°C in a refrigerator for GC–IMS analysis. Animal experiments were approved by the Animal Care and Use Committee of Liaocheng University (2023022706).

### 2.2. GC–IMS

The VOCs in the donkey meat samples were analyzed using a FlavourSpec® (Gesellschaft für Analytische Sensorysteme GmbH, G.A.S., Dortmund, Germany) GC–IMS unit equipped with a capillary column (MXT-5, 15 m × 0.53 mm × 1.0 μm) and an automatic headspace sampling unit (CTC-PAL, CTC Analytics AG, Zwingen, Switzerland).

A meat sample (1.5 g) was placed into a 20-mL-headspace glass bottle and incubated at 60°C for 15 min with spinning at 500 rpm. Subsequently, 500 μL of the headspace gas was automatically injected into the apparatus. The temperature of the injector was set to 85°C. The GC column temperature was 40°C with ≥99.999% purity nitrogen used as the carrier gas. The programmed flow of the carrier gas was 0–2 min, 2 mL/min; 2–10 min, 2–20 mL/min; 10–20 min, 20–100 mL/min. The 9.8-cm long drift tube and drift temperatures of the IMS instrument were 60°C and 45°C, respectively. The voltage of the drift tube was set at 5 kV. Drift gas was ≥99.999% purity nitrogen at a flow rate of 150 mL/min. 3H ionization was performed in positive ion mode.

### 2.3. VOCs identification

The retention indices (RIs) of the volatiles were compared with those of C4–C9 *n*-ketones (Sinopharm Chemical Reagent Beijing Co., Ltd., China) obtained under the same analytical conditions. The RIs and drift times (DTs) of the standards in the NIST (National Institute of Standards and Technology, Gaithersburg, MD, United States) 2014 library and GC–IMS database (G.A.S., Dortmund, Germany) were used to identify the VOCs.

### 2.4. Statistical analysis

Each sample was analyzed in triplicate and the data are represented as mean ± standard error of mean (SEM). Results were analyzed using SPSS version 24.0 (SPSS Inc., Chicago, IL, United States) with one-way analysis of variance (ANOVA) and Tukey’s test to evaluate the differences among them. *p* < 0.05 was regarded as significant. The spectra and fingerprints were processed using the Reporter plug-in and Gallery Plot plug-in, respectively. PCA, partial least squares discriminant analysis (PLS-DA), orthogonal PLS-DA, and heatmap analysis were performed using MetaboAnalyst 5.0 online software.^1^ Differential VOCs were determined according to a variable importance in projection (VIP) of >1 and *p* < 0.05.

## 3. Results

### 3.1. VOC profiles of donkey meats

**Figure 1:**
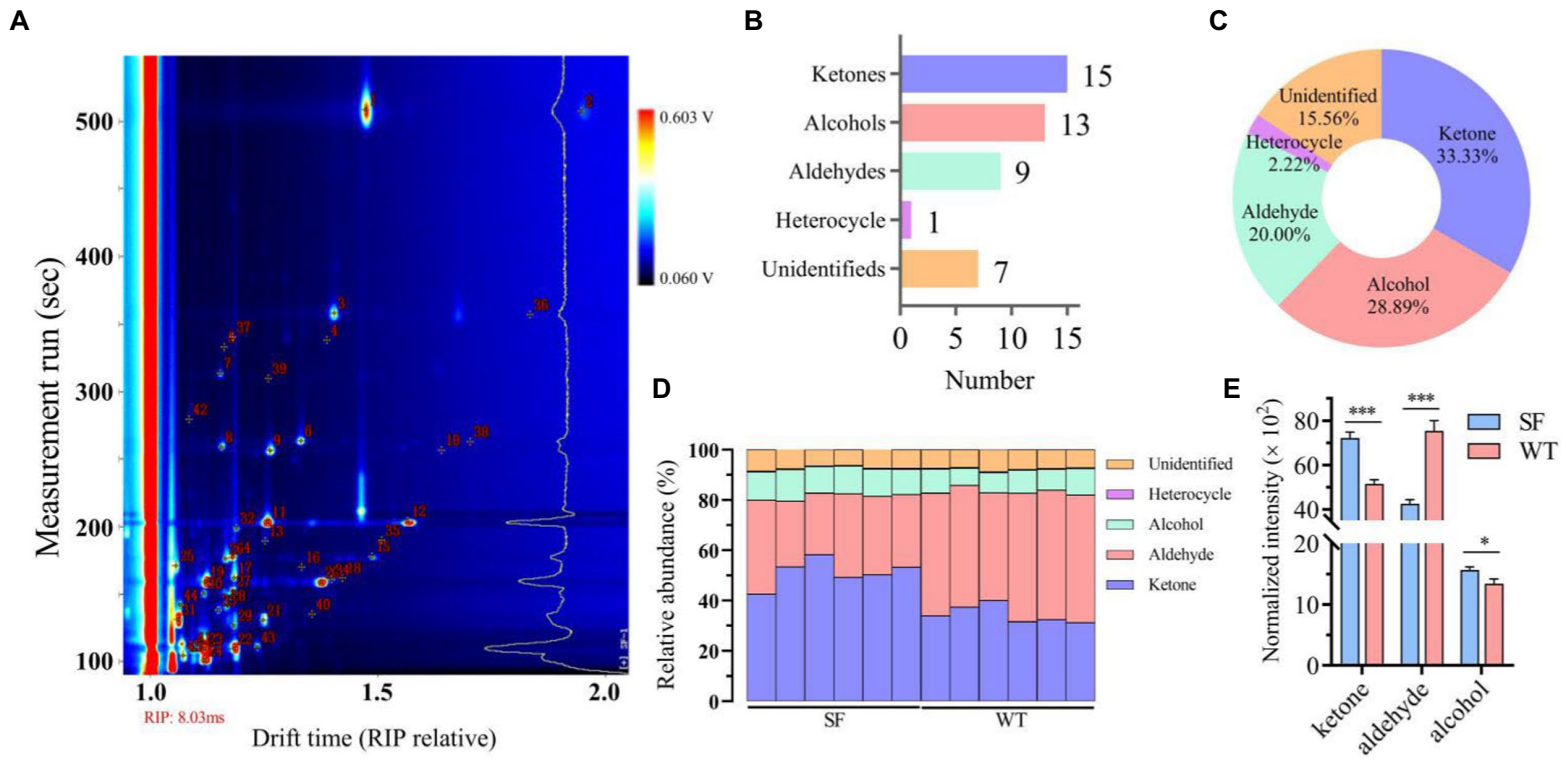
VOC profiles of donkey meats from two strains. Number of volatile compounds **(A)**. Number **(B)** and percentage **(C)** of volatile compound categories. Percentages **(D)** and concentrations **(E)** of volatiles compound typed in donkey meats from the two strains. Data presented as mean ± SEM (*n* = 6), *p < 0.05, ***p < 0.001. SF, Sanfen; WT, Wutou.

As shown in Figure 1 and Table 1, 45 VOCs were detected (15 ketones, 13 alcohols, 9 aldehydes,1 heterocycle, and 7 unidentified, i.e., 33.33% ketones, 28.89% alcohols, 20.00% aldehydes, 2.22% heterocycles, and 15.56% unidentified), 38 of which were identified in both strains. Thus, ketones, aldehydes, and alcohols are the most abundant VOCs in donkey meat (Figure 1D). Ketones and alcohols are significantly more abundant in SF than in WT donkeys (*p* < 0.001; *p* < 0.05), whereas aldehydes show the opposite trend (*p* < 0.001; Figure 1E).

**Table 1:** Information on VOCs in donkey meat.

No.	Compound	CAS#	Formula	MW	RI	Rt [sec]	Dt [a.u.]	Comment
1	Nonanal-M	C124196	C_9_H_18_O	142.2	1109.6	508.607	1.47485	Monomer
2	Nonanal-D	C124196	C_9_H_18_O	142.2	1108.5	507.026	1.94891	Dimer
3	Octanal-M	C124130	C_8_H_16_O	128.2	1004.8	357.807	1.40513	Monomer
4	3-Octenal	R286265	C_8_H_14_O	126.2	987.4	338.137	1.38963	
5	Oct-1-en-3-ol	C3391864	C_8_H_16_O	128.2	981.1	332.826	1.16454	
6	Heptanal-M	C111717	C_7_H_14_O	114.2	899.6	263.244	1.33256	Monomer
7	Benzaldehyde	C100527	C_7_H_6_O	106.1	958.7	313.704	1.15503	
8	Cyclohexanone	C108941	C_6_H_10_O	98.1	894.6	258.995	1.1582	
9	2-heptanone-M	C110430	C_7_H_14_O	114.2	891.1	256.339	1.2644	Monomer
10	2-heptanone-D	C110430	C_7_H_14_O	114.2	891.1	256.339	1.64167	Dimer
11	Hexanal-M	C66251	C_6_H_12_O	100.2	792.0	203.223	1.25965	Monomer
12	Hexanal-D	C66251	C_6_H_12_O	100.2	793.0	203.755	1.56717	Dimer
13	Pentan-1-ol-M	C71410	C_5_H_12_O	88.1	760.1	189.295	1.25375	Monomer
14	Methyl isobutyl ketone-M	C108101	C_6_H_12_O	100.2	730.7	177.37	1.18308	Monomer
15	Methyl isobutyl ketone-D	C108101	C_6_H_12_O	100.2	731.8	177.796	1.4879	Dimer
16	3-hydroxybutan-2-one-D	C513860	C_4_H_8_O_2_	88.1	712.3	169.917	1.33391	Dimer
17	Pentanal-M	C110623	C_5_H_10_O	86.1	692.4	161.825	1.18625	Monomer
18	Pentanal-D	C110623	C_5_H_10_O	86.1	692.4	161.825	1.42356	Dimer
19	2-Pentanone-M	C107879	C_5_H_10_O	86.1	683.2	158.844	1.12402	Monomer
20	2-Pentanone-D	C107879	C_5_H_10_O	86.1	683.2	158.844	1.37715	Dimer
21	2-Butanone-D	C78933	C_4_H_8_O	72.1	579.0	130.734	1.25058	Dimer
22	Unidentified 1	-	-	-	500.8	109.653	1.18519	
23	Acetone	C67641	C_3_H_6_O	58.1	500.5	109.553	1.12065	
24	Ethanol	C64175	C_2_H_6_O	46.1	468.4	100.888	1.12106	
25	3-hydroxybutan-2-one-M	C513860	C_4_H_8_O_2_	88.1	715.4	171.144	1.05908	Monomer
26	3-Methyl-3-buten-1-ol	C763326	C_5_H_10_O	86.1	727.3	175.977	1.16752	
27	1-butanol	C71363	C_4_H_10_O	74.1	657.6	151.923	1.1832	
28	2-methyl-1-propanol	C78831	C_4_H_10_O	74.1	618.4	141.369	1.17088	
29	Unidentified 2	-	-	-	563.8	126.642	1.18544	
30	Unidentified 3	-	-	-	651.2	150.205	1.11938	
31	2-Butanone-M	C78933	C_4_H_8_O	72.1	579.3	130.815	1.06228	Monomer
32	2-Hexanone	C591786	C_6_H_12_O	100.2	784.2	199.049	1.19104	
33	2-Butanol	C78922	C_4_H_10_O	74.1	604.8	137.687	1.15073	
34	Unidentified 4	-	-	-	690.0	160.869	1.40029	
35	Pentan-1-ol-D	C71410	C_5_H_12_O	88.1	761.8	189.973	1.50946	Dimer
36	Octanal-D	C124130	C_8_H_16_O	128.2	1004.0	356.763	1.83509	Dimer
37	6-methyl-5-hepten-2-one	C110930	C_8_H_14_O	126.2	989.8	340.229	1.18336	
38	Heptanal-D	C111717	C_7_H_14_O	114.2	898.9	262.688	1.70391	Dimer
39	(E)-hept-2-enal	C18829555	C_7_H_12_O	112.2	953.6	309.304	1.26154	
40	Unidentified 5	-	-	-	595.7	135.232	1.35706	
41	Isopropyl alcohol	C67630	C_3_H_8_O	60.1	501.5	109.845	1.09563	
42	Dihydro-2(3 h)-furanone	C96480	C_4_H_6_O_2_	86.1	918.5	279.358	1.08678	
43	Unidentified 6	-	-	-	504.7	110.683	1.23654	
44	Tetrahydrofurane	C109999	C_4_H_8_O	72.1	620.6	141.957	1.06659	
45	Unidentified 7	-	-	-	480.3	104.098	1.07416	

The VOCs were identified by retention index (RI) and drift time (Dt). MW, molecular weight; Rt, retention time.

### 3.2. Comparison of VOCs from the two strains

**Figure 2:**
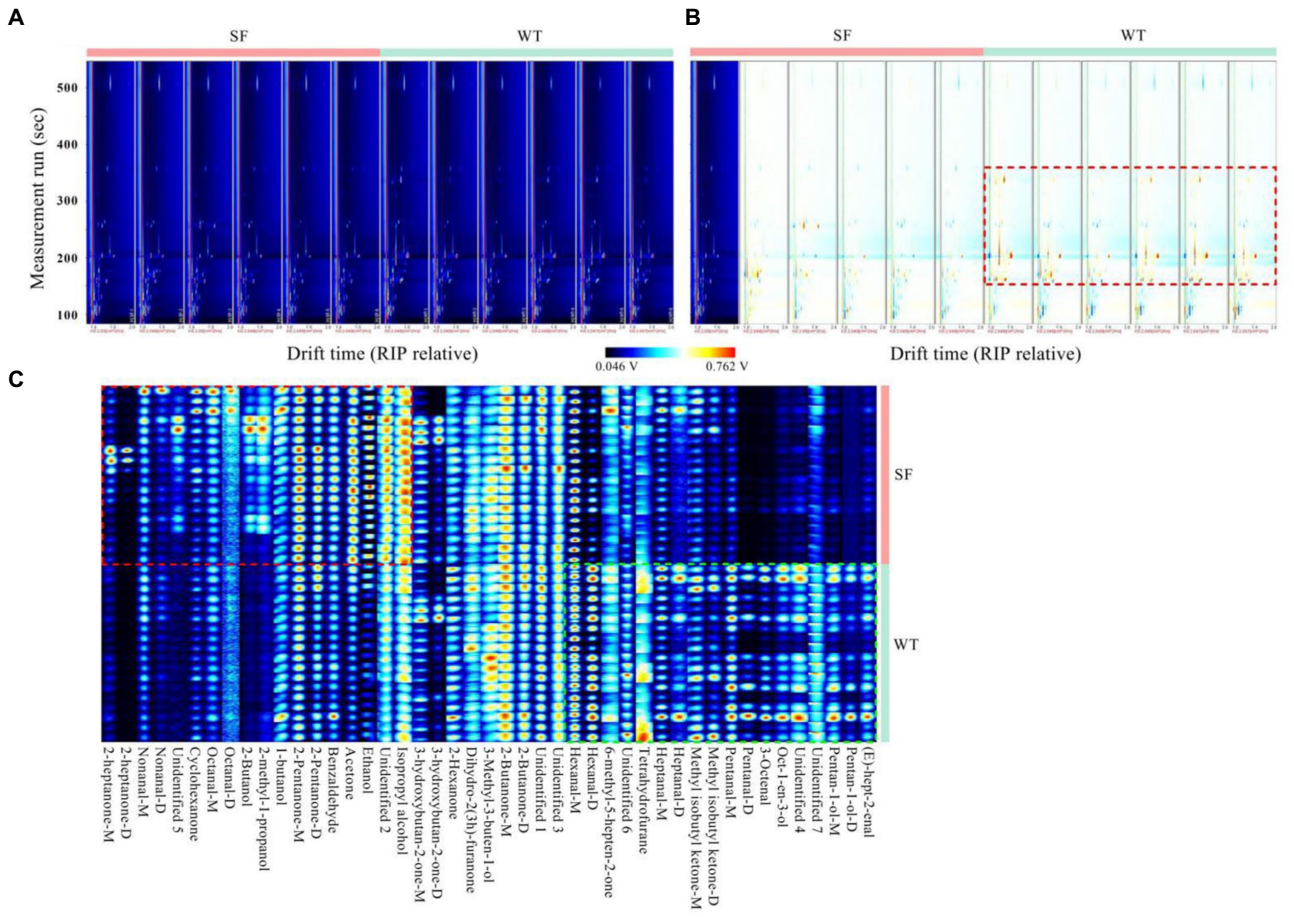
Comparison of the volatile components in donkey meats from two strains. Topographic representations of the spectra **(A)**, difference spectra **(B)**, and fingerprints of gallery plots **(C)** for volatile compounds. The brighter the color of the signal peak, the higher the concentration of the component. SF, Sanfen; WT, Wutou.

As shown in Figure 2A, the results show good repeatability according to topographic plots. Significant difference was observed between the fingerprints of the two strains (Figure 2B). In addition, isopropyl alcohol, ethanol, acetone, benzaldehyde, 2-pentanone-d, 2-pentanone-m, oct-1-en-3-ol, 3-octenal, pentanal-d, pentanal-m, methyl isobutyl ketone-d, methyl isobutyl ketone-m, heptanal-d, and heptanal-m present different signals and thus represent differential components in the fingerprint regions of the donkey meats from the two strains (Figure 2C).

### 3.3. Multivariate analysis of VOCs

**Figure 3:**
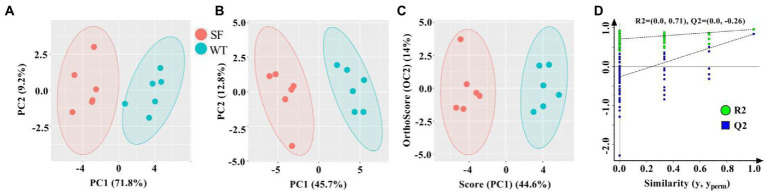
Multivariate analysis of the volatile components in meats from two donkey strains. Principal component analysis (PCA) **(A)**, partial least squares discriminant analysis (PLS-DA) **(B)**, and orthogonal PLS-DA **(C)** score plots based on flavoromics data. Corresponding OPLS-DA validation plots **(D)**. SF, Sanfen; WT, Wutou.

To better present and distinguish the differences between the donkey meats from the two strains, PCA, PLS-DA, and OPLS-DA analysis was performed (Figure 3). As shown in Figure 3A, the donkey meat samples are well differentiated according to strain using PCA, which is consistent with the results of PLS-DA and OPLS-DA (Figures 3B,C). Figure 3D shows R^2^ and Q^2^ intercept values are (0, 0.71) and (0, −0.26). All the Q^2^ points are lower than the rightmost original Q^2^ point, and the Q^2^ regression line is less than 0 at the intersection of the vertical coordinates, indicating that the OPLS-DA model is robust, reliable, and free from over-fitting. These results indicate that the strains of donkey meat can be well differentiated according to their VOCs using multivariate analysis.

**Figure 4:**
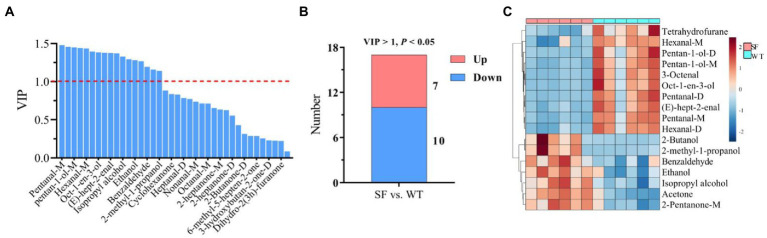
Differential volatile components between donkey meats from two strains. Variable importance in projection (VIP) of volatile components **(A)**. Number of significantly different volatile components **(B)**. Heatmap analysis of differential volatile components **(C)**. SF, Sanfen; WT, Wutou.

The VIP results for OPLS-DA are shown in Figure 4A, revealing that 17 differential VOCs were identified in donkey meats from the two strains by applying a criteria of VIP > 1. A total of 7 downregulated and 10 upregulated VOCs were identified for SF compared with WT by setting VIP > 1 and *p* < 0.05 (Figure 4B). These differential VOCs belong to four categories: 7 alcohols, 7 aldehydes, 1 heterocycle, and 2 ketones (Table 2). The levels of tetrahydrofurane, hexanal-m, pentan-1-ol-d, pentan-1-ol-m, 3-octenal, oct-1-en-3-ol, pentanal-d, (e)-hept-2-enal, pentanal-m, and hexanal-d are significantly lower for SF than for WT (*p* < 0.05), whereas 2-butanol, 2-methyl-1-propanol, benzaldehyde, ethanol, isopropyl alcohol, acetone, and 2-pentanone-m show the opposite trend (*p* < 0.05, Figure 4C; Table 2).

**Table 2:** Different volatile components of donkey meats from two strains (normalized intensity).

No.	Compound	Class	SF	WT	*P-*value	VIP
1	Pentan-1-ol-M	Alcohol	73.36 ± 7.58	319.54 ± 35.14	0.0001	1.450
2	Oct-1-en-3-ol	Alcohol	22.16 ± 1.38	75.28 ± 9.09	0.0003	1.385
3	Isopropyl alcohol	Alcohol	93.61 ± 1.49	80.60 ± 1.47	0.0009	1.330
4	Pentan-1-ol-D	Alcohol	15.58 ± 0.76	54.29 ± 9.11	0.0016	1.292
5	Ethanol	Alcohol	906.74 ± 37.05	513.16 ± 47.02	0.0018	1.282
6	2-Butanol	Alcohol	127.17 ± 15.87	42.22 ± 1.30	0.0081	1.157
7	2-methyl-1-propanol	Alcohol	96.38 ± 11.15	35.71 ± 1.91	0.0096	1.140
8	Pentanal-M	Aldehyde	249.02 ± 13.7	664.06 ± 52.47	0.0000	1.479
9	Hexanal-D	Aldehyde	812.67 ± 113.60	2934.29 ± 240.86	0.0000	1.458
10	Hexanal-M	Aldehyde	1058.54 ± 51.72	1485.86 ± 25.32	0.0001	1.434
11	3-Octenal	Aldehyde	69.14 ± 6.80	437.82 ± 61.65	0.0002	1.397
12	Pentanal-D	Aldehyde	16.85 ± 1.89	196.58 ± 36.96	0.0003	1.379
13	(E)-hept-2-enal	Aldehyde	38.30 ± 1.94	93.61 ± 9.20	0.0004	1.377
14	Benzaldehyde	Aldehyde	86.25 ± 2.17	69.29 ± 3.54	0.0054	1.195
15	Tetrahydrofurane	Heterocycle	32.55 ± 1.21	45.64 ± 2.01	0.0022	1.269
16	Acetone	Ketone	2625.99 ± 90.43	1248.76 ± 93.31	0.0001	1.441
17	2-Pentanone-M	Ketone	862.19 ± 17.49	657.34 ± 18.25	0.0004	1.371

SF, Sanfen; WT, Wutou; VIP, variable importance in projection.

## 4. Discussion

In this study, the VOCs in donkey meat were identified and analyzed by GC–IMS with chemometrics analysis. Accordingly, 38 VOCs belonging to four categories were identified in raw donkey meat. This is significantly lower than the number of VOCs previously identified by GC–MS in cooked donkey meat and cooked horse meat (11, 21). It is well known that VOCs are more abundant in cooked meat and that they are generated by the Maillard reaction between amino compounds and reducing sugars, lipid degradation, and lipid-Maillard interactions during heating (22). The present study has shown that ketones, alcohols, and aldehydes are the most abundant VOCs in uncooked donkey meat and that aldehydes are the characteristic VOCs in WT donkey meat. This is consistent with previous GC–MS results for Xiaohei donkey meat (3). The levels of ketones, alcohols, and aldehydes in SF and WT donkey meats are significantly different, with aldehydes being more abundant in WT donkey meat. Previous studies have demonstrated that the concentrations of different types of VOCs differ between breeds of pork and chicken (23, 24).

The GC-IMS spectra and fingerprints visually represent the results of flavor measurement between samples, where the fingerprint can be used to intuitively and quantitatively compare the differences in VOC profiles between samples (15). In the present study, significant differences were observed in the spectra of SF and WT donkey meats. These results were confirmed by their VOC fingerprints, where ethanol and hexanal-d show very clear differences between the two strains. This indicates that differences between SF and WT donkey meat samples can be quickly identified by GC–IMS analysis through their VOC profiles, which is in agreement with previous studies on pork from different pig breeds (18). Multivariate analysis, including unsupervised pattern recognition (UPR) and supervised pattern recognition (SPR), is used to analyze omics data from different experimental groups (25). UPR (PCA and hierarchical cluster analysis) is used to analyze data without grouping samples, which ignores random errors in the group (26). SPR includes PLS-DA and OPLS-DA analysis, which eliminates the deficiency of UPR, i.e., easy overfitting (27). Permutation validation is applied in OPLS-DA analysis to determine the overfitting of a model (28). In the present study, multivariate analysis methods (PCA, PLS-DA, OPLS-DA, and heatmap analysis) were used to confirm the accuracy of the data and fingerprints obtained by GC–IMS. The VOCs of donkey meats from the different strains were well differentiated using PCA, PLS-DA, and OPLS-DA analysis, and OPLS-DA eliminated over-fitting in the current study. A total of 17 differential VOCs were identified between the different strains, including hexanal-m, 3-octenal, oct-1-en-3-ol, hexanal-d, ethanol, isopropyl alcohol, and acetone, which is consistent with the fingerprint results. These results demonstrate that multivariate analysis can be applied to distinguishing different samples and screening biomarkers from GC–IMS data.

Previous studies have shown that the VOCs in meat are breed-dependent (29). This is consistent with the present study, in which the VOCs in donkey meat were also found to be strain-dependent. In this study, tetrahydrofurane, hexanal-m, pentan-1-ol-d, pentan-1-ol-m, 3-octenal, oct-1-en-3-ol, pentanal-d, (e)-hept-2-enal, pentanal-m, and hexanal-d were identified as characteristic VOCs of WT donkey meat, whereas 2-butanol, 2-methyl-1-propanol, benzaldehyde, ethanol, isopropyl alcohol, acetone, and 2-pentanone-m were identified as characteristic VOCs of SF donkey meat. This is consistent with the findings reported for Xiaohei donkey meat (3). The formation of VOCs is complex and their sources are extensive, among which, lipids are the main sources of meat flavor development. Lipid degradation is the basis for the formation of unique meat flavor compounds such as aldehydes, ketones, and alcohols (30). 18:1 fatty acid is oxidized to produce octanal, nonanal, and 2-undecenal; 18:2 fatty acid is oxidized to produce hexanal, 2-nonenal, (E,E)-2,4-decadienal, and 2-pentylfuran; and 20:4 fatty acid is oxidized to produce heptanal, 1-octene-3-ol (31). Recent research has shown that the fatty acid compositions of SF and WT donkey meats are not significantly different, and 37 different lipid molecules were identified between them, mainly including glycerolipids and glycerophospholipids (32). Fatty acids at different positions of lipid molecules also affect the formation of VOCs. For example, fatty acids in glycerophospholipids at the sn-2 site show high thermal oxidative stability, whereas triglyceride is more readily hydrolyzed to free fatty acids and then oxidized to form VOCs (33, 34). In addition, VOCs can undergo further degradation into other VOCs, as observed in different types of meat (35).

## 5. Conclusion

In the present study, VOCs in donkey meats from different strains were comprehensively analyzed and compared using GC–IMS. Overall, 38 VOCs belonging to four different compound categories were identified. The donkey meats from the two strains can be differentiated by using their VOC fingerprints and multivariate analysis, with 17 different VOCs being identified as potential markers to distinguish different donkey meat strains. To conclude, our results may facilitate a better understanding of the characteristic VOCs of donkey meat and provide a novel strategy for its authentication. However, the characteristic VOCs of donkey meat are not clear and require further studies.

## Data availability statement

The original contributions presented in the study are included in the article/supplementary material, further inquiries can be directed to the corresponding authors.

## Ethics statement

The animal study was reviewed and approved by Animal Care and Use Committee of Liaocheng University.

## Author contributions

LM: conceptualization, methodology, investigation, data curation, and writing-original draft and editing. WR: formal analysis, data curation, and investigation. MS, YD, HC, and HQ: data curation, investigation, and resources. WC, MZ, and GL: investigation and data curation. CW and ML: supervision, writing-review and editing, and project administration. All authors contributed to the article and approved the submitted version.

## Funding

This work was supported by the Shandong Provincial Natural Science Foundation (ZR2022QE143 and ZR2022QC130), the Shandong Province Modern Agricultural Technology System Donkey Industrial Innovation Team (SDAIT-27), the Scientific Research Fund of Liaocheng University (318052019 and 318052057), the Open Project of Liaocheng University Animal Husbandry Discipline (319462207-10), the Livestock and Poultry Breeds Project of Ministry of Agriculture and Rural Affairs (19211162), the Shandong Rural Revitalization Science and Technology Innovation Action Plan (2021TZXD012), and the Innovation and Entrepreneurship Training Program for College Students (CXCY2022176 and CXCY2022381).

## Conflict of interest

The authors declare that the research was conducted in the absence of any commercial or financial relationships that could be construed as a potential conflict of interest.

## Publisher’s note

All claims expressed in this article are solely those of the authors and do not necessarily represent those of their affiliated organizations, or those of the publisher, the editors and the reviewers. Any product that may be evaluated in this article, or claim that may be made by its manufacturer, is not guaranteed or endorsed by the publisher.

## Footnotes

^1^ https://www.metaboanalyst.ca/
